# The dispositions of things: the non‐human dimension of power and ethics in patient‐centred medicine

**DOI:** 10.1111/1467-9566.12431

**Published:** 2016-07-27

**Authors:** John Gardner, Alan Cribb

**Affiliations:** ^1^ Science and Technology Studies UnitDepartment of SociologyUniversity of YorkYorkUK; ^2^ Department of Education and Professional StudiesKings College LondonLondonUK

**Keywords:** actor‐network theory, empirical ethics, bioethics, shared decision‐making

## Abstract

This article explores power relations between clinicians, patients and families as clinicians engage in patient‐centred ethical work. Specifically, we draw on actor‐network theory to interrogate the role of non‐human elements in distributing power relations in clinical settings, as clinicians attempt to manage the expectations of patients and families. Using the activities of a multidisciplinary team providing deep brain stimulation to children with severe movement disorders as an example, we illustrate how a patient‐centred tool is implicated in establishing relations that constitute four modes of power: ‘power over’, ‘power to’, “power storage” and “power/discretion”. We argue that understanding the role of non‐human elements in structuring power relations can guide and inform bioethical discussions on the suitability of patient‐centred approaches in clinical settings.

## Introduction

Dystonia is a disorder caused by abnormal activity in the basal ganglia area of the brain. It is characterised by uncontrolled, sustained and intermittent muscular contraction, which in severe cases can result in painful, crippling body postures. Medications provide relief for some sufferers, but for others, particularly those with severe dystonia, medications are ineffective or the side‐effects are intolerable. Indeed, dystonia was recently identified as one of several neurological disorders for which there is an urgent need for new, more effective therapies (Nuffield Council on Bioethics 2013).

There is hope among clinicians and sufferers that deep brain stimulation (DBS) will prove to be such a therapy. DBS involves the use of a pacemaker‐like device to deliver constant electrical stimulation to areas deep in the brain. The considerable effectiveness of DBS in managing the symptoms of Parkinson's disease has led clinicians to explore DBS as technique for managing other neurological disorders, including dystonia. Worldwide, over a thousand individuals with predominantly primary dystonia have received DBS. Many have experienced useful functional improvements and some have experienced dramatic improvements. Yet, due to the novelty of the technique and uncertainties over its long‐term safety, DBS for dystonia is considered to occupy a realm between experimental and routine clinical therapy (Nuffield Council on Bioethics 2013). Recently, a few clinical services have been established to deliver DBS specifically to children and young people with dystonia, including those with complex secondary dystonia who, due the presence of other neurological pathologies, tend to respond more modestly to the technique than those with pure (primary) dystonia (Marks *et al*. 2009).

This article explores the activities of one of these teams as it manages the expectations of patients and families. The team, which we refer to as the Paediatric Motor Disorder Service (PMDS), is based at a children's hospital in the UK. It has a multidisciplinary structure and includes neurologists (one of whom was a clinical research fellow), a specialist nurse, an occupational therapist (OT), physiotherapists (PT), a speech and language therapist, a clinical psychologist and an administrator. This structure of the team, and indeed many aspects of the context in which they work, reflect their commitment to patient‐centred health care. The team, for example, adopts a biopsychosocial perspective of disease, according to which illness is seen as having social and psychological dimensions that need to be recognised and accommodated during clinical interactions. The hospital within which the team works was specifically designed to encourage comprehensive interdisciplinary care for families (see Gardner 2016).

Managing the expectations of patients and families is a key challenge for the PMDS. DBS has been the subject of media‐generated hype (Gilbert and Ovadia 2011, Racine *et al*. 2007) and partly because of this many families that arrive at the PMDS have high expectations about what DBS can offer; expectations that team members feel are unrealistic. Elsewhere, we have argued that the management of patient expectations by clinicians is an important part of biomedical innovation more generally (Gardner *et al*. 2015). In this article we examine the team's attempts to negotiate expectations with patients and families, which we recognise as a form of ethical work that is both responsive to patient and family agendas and, at the same time, increases the alignment between family and team perspectives. We carefully examine the goal‐setting interactions through which this ethical work is conducted, focusing particularly on the deployment and enactment of power relations between the clinicians, patients and families. Specifically, we examine the role of non‐human elements in managing and deploying power relations and enacting ethics. In the process of managing expectations, team members utilise a patient‐centred tool they have adopted from occupational therapy. We draw on observational and interview data to illustrate how this tool designates relations that constitute the modes of power delineated by John Law (1991a). These include ‘power over’, ‘power to’, ‘power storage’ and ‘power/discretion’. Through this account we hope to make two important contributions. Firstly, by drawing attention to the implicit normativity in patient‐centred tools and by exploring patient‐centred medicine in practice, we shed light on how the movement is implicated in redrawing and reconfiguring power relations in clinical settings. Secondly, in so doing, we highlight the implications for social scientists and bioethicists interested in identifying and facilitating good ethical work.

## Power and the patient‐centred medicine movement

In many countries having a greater level of patient involvement in health care has become a policy imperative (Thompson 2007). This is particularly so in the UK, in which successive reports and statements in the health policy arena have stressed the need for patient empowerment that, it is argued, can help improve clinical outcomes and led to greater efficiencies in healthcare systems (Department of Health 1999, 2003, National Health Service [NHS] 2013). Similarly, an emerging body of work in the health sciences has endorsed the movement towards patient‐centredness in health care. Patient‐centredness is seldom clearly defined but commentators have suggested that it is generally equated with several key features (Mead and Bower 2000). These include the promotion of a biopsychosocial approach to understanding health and illness as a means of mitigating the perceived shortcomings of the biomedical model; the championing of practices that recognise and accommodate the patient‐as‐person, in which the unique biography of patients and their perceptions of their illness are taken into account during clinical decision‐making; and, following from this, a movement towards greater patient participation in clinical decision‐making (Mead and Bower 2000).

Calls for greater patient empowerment can be seen as a reflection of wider sociopolitical trends, such as the valorisation of individual choice and consumerism (Thompson 2007) and increased layperson scepticism toward professional authority. Generally patient‐centredness and patient empowerment are framed as a rejection of the medical paternalism of the past, in which the clinician was presumed to understand what was in the patient's interests, and in which the authority of the clinician was paramount. Patient empowerment and patient‐centredness are, in other words, positioned as a needed rebalancing of the power dynamic between the clinician and the patient; a move from clinical interactions in which the clinician has power over the patient towards a more egalitarian dynamic. Patients and family members should be provided with a space to be active participants and have their concerns heard, and health care practices should, it is argued, be reoriented so that such concerns are accommodated.

Cribb has noted that there is a gap between the ideal of patient empowerment as it is promoted in health policy and the health science literature, and actual healthcare practice. It is, Cribb suggests, very difficult to translate the principle into practice, as it requires forging new habits, routines and protocols in settings that may already be under considerable strain. Hesitancy among some professionals may reflect legitimate ethical concerns about the contribution of patients in particular settings (Cribb 2011). Additionally, given the vast variation in healthcare contexts, it is likely that what constitutes a desirable enactment of patient‐centredness and patient empowerment will differ according the nature of the illness and the socio‐technical characteristics of the clinic settings. With this in mind, and with the intention of anticipating the ethical implications of patient‐centredness and patient empowerment, we explore a specific clinical context in which a patient‐centred approach has been adopted. Entwistle and Watt (2006) have argued that any appraisal of patient‐centredness and patient involvement should address the following points:

What kinds of activities should individuals be involved in? What kinds of relationships and people should individuals be involved with? Who are we imagining are the agents of empowerment? i.e. Who is supposed to initiate or support involvement for whom? (Cribb 2011: 11)

The premise of this article is that, in order to address these questions and thus identify those forms or patient involvement that might be most appropriate in a given setting, it is useful to explicitly address configurations of power, and it is necessary to explore how tools and non‐human objects are implicated in these configurations of power.

Several sociological studies have examined the enactment of patient‐centredness in clinical settings (Dubbin *et al*. 2013, Gardner 2016, Liberati *et al*. 2015). Using a Bourdieusian analytical frame, Dubbin *et al*. (2013) explored the role of human agents’ cultural resources and dispositions in clinical practice and noted that patient‐centred interactions were often characterised by an asymmetrical power dynamic, in which the clinician was able to encourage particular patient behaviour. We interrogate this power dynamic in depth, and we adopt the position of Liberati *et al*. (2015) that the enactment of patient centredness has an important material, non‐human dimension. Indeed, in this article we illustrate how a particular patient‐centred tool known as the Canadian occupational performance measure (COPM) structures the activities of individuals in clinical settings and how it is implicated in initiating and supporting particular modes of patient involvement. Such tools are actively structuring power relations and are engaged in forms of ethical work, which may or may not be desirable, in other clinical settings.

Specifically, in this article we revive Law's taxonomy of power (1991a) and deploy it as an actor‐network theory ANT‐inspired analytical lens. ANT theorists have argued that power should not be seen as a thing in‐itself and it should not be used as an explanans in its own right. Rather, power should be seen as a relational effect; as the consequence of specific relations between entities that are brought about via ordering and assembling practices (Latour 2005, Law 1986, 1991b). In light of this tenet Law has proposed four modes of power. Firstly, there is ‘power over’*,* which describes circumstances in which a powerful agent imposes its interests upon others in such a way that may be contrary to the interests of the latter (Law 1991a: 168). It is this mode of power that has been said to characterise paternalistic medical authority. The second mode, which is not necessarily distinct from the first, is ‘power to’*,* and refers to the way in which agents are enabled and empowered via their immersion in a socio‐technical collective (Law 1991a: 167). For example, agents may strategically arrogate and deploy dominant medical discourses in a process of self‐affirmation (Rabinow 2008). This parallels what Foucault referred to as the productive capacity of power, and it also aligns with Foucault's characterisation of power as being fluid and infused throughout all social relations (Foucault 1991). Law argues that this fluidity does not exclude the potential for more rigid power arrangements, however. Hence, the third mode is ‘power storage’. This refers to circumstances in which socio‐technical relations and thus, their ‘power over’ effects and/or ‘power to’ effects, have acquired a degree of stability (Law 1991a: 168–9). Agents, for example, may become embedded in resilient socio‐technical networks with highly asymmetrical power dynamics: such situations constitute a state of domination. An example of this is Latimer's (1997) account of an acute medical unit in which people were configured as particular types of patients via organisational routines, which thus had the effect or producing and reproducing social hierarchies and divisions. And fourth, agents in socio‐technical networks, particularly those who benefit from such arrangements, possess the capacity to refrain from action, and choose between alternative actions which may each have their own ‘power over’ consequences and ‘power to’ consequences. This is ‘power/discretion’ (Law 1991a: 170–1). Agents do not necessarily act in a mechanistic fashion, therefore, but the degree to which they can resist, and the degree to which they can exercise ‘power/discretion’*,* can vary significantly between agents and between contexts.

In any given context, then, an agent is immersed within, and configured by, socio‐technical relations with ‘power over’ and ‘power to’ effects that, depending on the stability of the socio‐technical relations, can be stored and can be deployed with various degrees of discretion (Law 1991a: 172). Adopting this characterisation of power as an analytical lens leads to particular avenues of inquiry: how are the particular socio‐technical relations that constitute power assembled and brought together in the first place? What specific set of relations constitute ‘power over’ and or ‘power to’, which entities or agents are affected, and how are these relations made durable? It is these questions that guide this exploration of the PMDS team's attempt to manage the expectations of their patients As a way of interrogating the effects of patient‐centred practices in the PMDS (and thus as a way of anticipating the impact of the patient‐centred movement as a whole), we illustrate how the four modes of power are manifest during the course of the goal‐setting session.

In doing so, we adopt an ontological tenet of ANT and, indeed, much of science and technology studies (STS). Traditionally, sociological inquiry and ethnographic methodology have tended to prioritise the interactions of human actors. In effect, the social was restricted to human interaction and intersubjective meanings (Latour 2005: 65). Yet the division between human as agents and non‐humans as inert has been challenged by ANT and STS scholars (see Callon 1986, Latour 1987, Mol 2002). These theorists have argued that non‐human elements have the capacity to prompt, constrain, enable, and transmute human action in ways that cannot be reduced to the intentions of other humans. Non‐human elements do not determine human action, as they inevitably possess a degree of flexibility, enabling them to be adapted to local cultural understandings. But they do, however, have an obduracy which shapes and constrains the practices that they enable (Akrich 1992). In other words, non‐human elements are key components of the socio‐technical relations that generate power effects. In light of this, we focus specifically on the PMDS team's use of the patient‐centred COPM during their goal‐setting sessions with patients and families, and we explicitly examine how the tool itself is implicated in the generation of power effects during the interaction.

This has important implications for bioethicists and others interested in analysing would‐be ethical improvements in healthcare policies or practices. First, it provides one model for moving beyond the principle advocacy of ethical aspirations towards the characterisation of what might be entailed by their social enactment or embodiment. Second, it indicates how this focus on ethical enactment needs to look beyond human agency and dispositions so as to encompass attention to the agency and dispositions of non‐human elements.

## Methodology

Data for this article were collected during a 12‐month study of the PMDS using ethnographic methods. The purpose of the fieldwork was to identify the challenges associated with the implementation of a novel technology (DBS) in a new clinical service (the PMDS). The researcher (JG) sought to identify what PMDS team members felt were the major challenges in providing a DBS service, and how team members manage these in their day‐to‐day clinical work. Several data collection methods were used. First, JG observed weekly PMDS team meetings (*n = *31), during which team members articulated key challenges, one of which was managing the expectations of patients. Second, specific interactions in which team members encountered and managed these challenges were observed (*n = *6). This included goal‐setting sessions in which team members attempted to manage the expectations of the patients and families. During all observations, JG attempted to record, in handwritten notes, the discussions and actions of the participants, as well as the key aspects of the material context. John Zeisel's (1984) recommended method for recording the material context was used. This involved creating annotated schematics of the space within which the interaction took place; noting how objects were used to create personalised or professional space; noting how objects and the material terrain where adapted for specific activities; and noting how props and objects (such as signs) were used to communicate how a space should be used. These aspects can provide some insight into how participants mould and utilise non‐human objects and their material context, and how these are subsequently implicated in shaping human action (Zeisel 1984).

Third, PMDS team members were interviewed (*n = *12), during which they were prompted to reflect upon their practices for managing day‐to‐day challenges. JG used these as an opportunity to raise specific queries from his observations and to ask the participants about their use of various non‐human elements and their material context during the clinical interactions. All interviews were audio‐recorded, and together with the observation notes, were coded using NVivo software according to key themes relating to the challenges of implementing DBS in a new clinical service. Some of these themes were presented to the team members during their annual away day, providing JG with valuable feedback.

A NHS Research Ethics Committee granted ethical approval for the fieldwork. Informed consent was obtained from all participants (PMDS team members, patients and supporting family members) who were 16‐years old or older. Assent was obtained from those under 16‐years of age, and informed consent was obtained from supporting guardians. Participant leaflets for children and young people were modelled on the format provided by Alderson and Morrow (2011).

## The ethical challenge of managing expectations in DBS

It is common for the families of new referrals to arrive at the PMDS with what team members describe as unrealistic expectations about DBS. As the clinical research fellow explains:

The problem is the press reports the case studies that do well – so there can be perception that DBS will get my child to walk. (Interview)

However, while DBS may indeed enable a few patients to walk again, for most patients the benefits of DBS will be more modest. As the neurologist explained:

We tell families that we can help improve functions that the child already possesses to some degree. If they are not walking at all, then DBS will not enable them to walk. (Neurologist, team meeting field notes)

This problem of expectations is compounded by the need to be responsive to the desperation and vulnerability of DBS patients and families (Marks *et al*. 2009). In an era when facilitating patients’ autonomy and enhancing patients’ capacity for decision‐making are seen as fundamental to ethical medical practice, understanding and negotiating perceptions and hopes is important ethical work. Indeed, ethicists (Bell *et al*. 2009) have argued that conveying risks and benefits to vulnerable patients is one of the key ethical challenges associated with all DBS therapies.

### The goal‐setting session and the COPM

The multidisciplinary structure of the team has enabled them to respond pragmatically to challenges by drawing on tools and the capacities and expertise of several professions. By adopting a specific tool from occupational therapy and utilising the skill‐sets of team members, they have developed a novel strategy – a goal‐setting session – for managing expectations. This session takes place after the pre‐surgical assessments have been conducted (during which the therapists obtain some idea of how the individual patient may benefit from DBS) and at least a month before the date of surgical implantation (providing patients and families with time to decide whether or not to proceed).

The session is organised around the COPM. The COPM is a standardised, semi‐structured interview developed by a team of Canadian OTs led by Mary Law in the 1980s. It was designed as a tool for therapists to measure and quantify changes in a client's perception of their functional abilities over time, such as before and after a therapeutic intervention. During the interview, clients are asked to identify five key tasks of daily living (such as washing the dishes and brushing teeth) that they would like to improve. For each task, they are then asked to rate, on a scale from one to ten, the importance of the task to them; their ability to perform that task and their satisfaction with their ability. From this set of numbers an overall average is calculated. In order to help maintain consistency (and ensure that scores can be compared), users of the COPM are provided with a manual containing a set of instructions and a standardised score sheet.

The COPM measures patients’ self‐perceptions of their abilities. According to the authors, the logic behind this focus on self‐perception is that the clinically important aspects of an affliction are the way it impacts on the actual, day‐to‐day life of the client (Law *et al*. 2005). We can see here how a particular normative orientation; an orientation that is a key aspect of patient‐centred medicine, has been incorporated into the tool. This is an orientation that attaches a heavy weighting both to the perceptions and values of patients and families and to broader functioning, rather than to measures of biomedical clinical signs. The tool is based on the belief that disease and treatment should be understood and assessed according to its impact on those aspects of a client's life that the client feels are important.

The team has adapted the COPM to fit local clinical exigencies. Patients are asked to identify the five key daily living tasks that are important to them very early on during their time with the PMDS, so that they can then attempt to perform these tasks as part of their pre‐surgical assessments. The COPM in its original form was intended to involve one therapist and one client. In the team version, the patient and supporting family members will be involved, and several team members will conduct the session together. Usually, this includes the OT and a physiotherapist (PT), who by the time of the session have an idea of how the individual patient may actually respond to DBS. Using Law's characterisation of power, we illustrate how the patient‐centred COPM is implicated in assembling and structuring power relations during the goal‐setting session. Specifically, we draw attention to the assigning of authority and spokespersons roles within the session, both of which entail instances of ‘power over’ and ‘power to’, and varying degrees of ‘power/discretion’.

### Negotiating expectations with the COPM

As with any clinical protocol (Berg 1998), the COPM embodies a script that delegates particular roles to those involved in session. It prescribes a particular arrangement of human and non‐human elements, and particular actions in space and time. Indeed, this is how it constitutes a form of ‘power storage’: each goal‐setting session follows roughly the same format as that instructed by the COPM manual, and thus the specific relations that constitute ‘power over’/’power to’ during the session have become embedded in the routine clinical practice of the PMDS.

Importantly for the PMDS, if the script is adhered to sufficiently closely, the five tasks of daily living will became the basis of five ‘realistic’ goals for the DBS intervention via a process of negotiation. In order to illustrate exactly how the tool is implicated in distributing power relations among agents, and how it leads to the formulation of five realistic goals, we will use field notes from an observation of a goal‐setting session involving Carl (pseudonym), a 16‐year‐old patient with secondary dystonia, and his mother.

### The therapists as leaders of the goal‐setting session: ‘power over’

The COPM delegates a leadership role to the therapists, reifying their professional authority during their interactions with patients and families. In this role they are accorded some ‘power to’ direct the arrangement of elements during the session so that it follows the COPM format, which in turn entails a degree of ‘power over’ the patients and families. The therapists are instructed to guide and direct patients and their family members throughout the interaction, prompting them to offer responses that are mandated by the COPM script. This role begins when the patients and families are first asked to identify five tasks of daily living that they would like to improve (which in the PMDS takes place prior to pre‐surgical assessments). The COPM manual instructs:

It is important that clients identify occupations that they want to do in daily life … . The therapist should encourage clients to think about a typical day and describe the occupations that they typically do. (Law *et al*. 2005: 13)

The leaders are required to guide the patients through the portion of the COPM score sheet that lists a number of possible areas of concern: personal care, functional mobility, community management, household management (cleaning and cooking), play/school, recreations, and socialisation (Law *et al*. 2005). These areas of concern are used to prompt the family to think about the impact of the patient's motor disorder on their day‐to‐day life, and for each area, families and patients are asked to identify specific tasks they would like to improve (such as dressing, hygiene, visiting friends and preparing food). The family is then asked to identify, from these, which five are most important. It is these five tasks that the patient will attempt to perform as part of the pre‐surgical assessments, and that the team will later use as the basis for negotiating expectations during the goal‐setting session.

The leaders are also expected to carefully arrange the space within which the session occurs. The space is arranged so that it appears informal and unintimidating, with the aim of facilitating honest, inclusive and open communication. Such arrangements are intended to ensure the active engagement of, but also the compliance, of patients and families during the session. Such arrangements are thus part of the relations that constitute both the clinicians constraining ‘power over’ the patients and families, and simultaneously, the patients and families’ constrained ‘power to’ engage in the session (the patient's ‘power to’ engage will be explored in more detail further on).

In the PMDS, the OT and the PT arrange the space in a large, quiet and private room so that all those involved in the session are seated in a circle. At the beginning of the session they restate the tasks that had previously been identified and then prompt the patient and family members to clarify the specific problem they are having with that task. The aim is to produce at least five clearly delineated tasks that can become the basis for setting clearly delineated goals:

OT Now, when I met you last time, we talked about the things that you wanted to improve. You identified a number of things … . These were handwriting, shaving, self‐feeding, drinking, and using public transport.

PT So with your handwriting, what aspects are you not happy with? Speed? accuracy?

Carl Both … I get hand cramps. I would like to be able to handwrite on a clear page without making a mark all over the page.

PT Okay, what about drinking?

OT I've noticed that when you drink from a bottle, you bring it to your mouth and tip your whole head back. Is that to make sure your arm doesn't flick it away and spill it?

Carl Yeah.

For each one of the five tasks, patients and their family members are prompted by the OT and PT to be as specific as possible; to specifically and explicitly outline what exactly they perceive the child's problem to be. Here we see an illustration of the clinicians’ constraining ‘power over’ the patient and that latter's constrained ‘power to’ engage in the session in a meaningful way. The COPM manual states:

It is essential that therapists use their skills in interviewing, probing for full responses. (Law *et al*. 2005).

The COPM format is thus by no means independent of human skills and dispositions, but rather both shapes and harnesses them. Indeed, the therapists’ ‘power over’ patients and families is partly a consequence of the deployment of such skills. Without them, the interaction may proceed in a way that is off‐script and will fail to generate the intended goals. In this case, as Carl and his mother are prompted to add more and more detail, each particular problem becomes more intelligible and actively delineated. In effect, the resulting five tasks are the product of an interaction between the therapists, the patient and the supporting family members, as scripted by the COPM.

### Disclosing predicted benefits using patient‐accessible frames of reference

At the same time that the five tasks are being clarified, the PT and OT express their predictions on how the patient's ability to carry out these tasks will be affected by DBS. These predictions are based on their observations from pre‐surgical assessments and their experience of previous DBS outcomes. If dystonia (involuntary movement) is deemed to be the cause of the problem, then the therapists will tentatively predict that there will be some improvement:

OT Carl, tell me about shaving. Why does mum do it for you?

Carl It pulls on my hair, it is really sore.

OT His arm pulls away and the hair gets caught in the shaver. It is definitely the involuntary movements that are making it difficult to shave . … Carl – if DBS does reduce your involuntary movements, you will find it easier to shave.

And, if a difficulty is perceived by the therapists to be caused by muscle weakness, contractures or spasticity, then they will predict that no improvement will occur. Here the OT discusses Carl's handwriting:

OT I think your computer is your best option. DBS may help a bit, but you won't be able to rely on your handwriting. We have noticed some, very minor improvements in patients, but that is after 4 or 5 years.

Once the patient and their family have been prompted to clarify the tasks they would like to improve and the therapists have disclosed their predictions, the COPM script requires that therapists guide the family towards a goal for each task. This involves negotiation, during which particular goals are delineated as realistic and others as unrealistic. Here is an example of the PT guiding and negotiating with Carl:

PT About this problem with stability on public transport. We noticed you have muscle weakness around your pelvis that DBS won't improve. You could probably improve it with a lot of hard work and exercise in the gym, but we shouldn't set a goal that you are not prepared to put in the effort for in the first place.

Carl I'm not motivated, but that is because it takes me so much energy to do things!

PT Can we agree that we don't put this as an initial goal? You could tackle it when you have some more motivation, but I don't think we should put it down as a goal for DBS. We should aim for other goals.

As a result of this negotiation, one potential goal has been discarded as unobtainable and thus unrealistic. The PT has used her COPM‐designated role as leader (along with her authority as a clinician) and the COPM‐scripted negotiation space (characterised by Carl's constrained ‘power to’ engage in the session) to define what is and what is not realistic, and to thus prompt Carl to agree on particular, obtainable goals. Below is another example, involving the psychologist:

Psy Carl, about your wish to have more confidence in public. We need to clarify: What would it take to improve your confidence? Would it be not falling at all? Or falling less?

Carl Just less falls and less jerky movements.

Psy So, would just a little bit of improvement, then, help with your confidence, do you think?

Carl Yes.

Psy Because some people might not be happy if they still had some visible signs of the movement disorder. It is good that you think that a little improvement will help.

Here, the psychologist has suggested to Carl that ‘a little improvement’ is a more suitable goal. If all goes to plan and all participants adhere to the script, the resulting five realistic goals for DBS will reflect the family's wishes for meaningful improvement in the patient's day‐to‐day functioning and will reflect the therapists’ educated (but tentative) predictions.

In this way the COPM mediates between the expectations of patients and the judgements of therapists: it delegates authority to the therapists and instructs them to prompt families to be explicit about their difficulties and hopes. The resulting prediction, then, arises from an interaction in which clinicians have been accorded a degree of ‘power over’ the patients and families, and in which the patients and families have a constrained ‘power to’ engage. Importantly, the COPM format prompts therapists to communicate the effects of DBS in terms that are comprehensible and meaningful to families. Some important ethical work is done here. For example, some aspects of patients’ and family members’ perspectives are acknowledged and treated as central; and information is disclosed to patients and families in a direct and accessible form. According to team members, the latter is a key advantage of the COPM‐scripted goal‐setting session:

This is the idea of doing these setting sessions … we're not nebulously talking about things getting better, we're talking about you being able to dress that right arm better. (Clinical research fellow, interview)

A well‐informed family, then, is the ideal product of an interaction that has been carefully coordinated according to the COPM. Patients and families are then given at least a month to decide whether or not they would like to proceed with DBS.

### Patients and family members as spokespersons

As Carl's example illustrates, a successful goal‐setting session entails an arrangement in which patients and families have ‘power to’ engage in the session. In the process of being instructed, guided and prompted during the goal‐setting session, patients and their family members are called upon to act as spokespersons. Spokespersons (or spokes‐things) are those elements that are designated with the authority to speak on behalf of or delineate and define some entity or state of affairs (Latour, 2005: 31). During goal‐setting sessions, patients and family members are designated to speak with some authority on the impact of the movement disorder on the patient's day‐to‐day life and to delineate which specific aspects of this impact are important. This is not to say that patients and families can say as they please: they are directed towards particular modes of expression required for the COPM to function – hence, we have referred to their *constrained* ‘power to’ engage during the session. The COPM script then, both authorises the importance of responsiveness to patients and also configures a particular patient voice.

The PMDS therapists use a range of tools and techniques to configure patients’ and family members’ constrained ‘power to’ engage in the session. Indeed, therapists will attempt to ensure that both the patient and supporting family are accorded this spokesperson role. It is common for patients to have goals and expectations different from those of their supporting family. For younger patients, for example, these revolve around being able to interact with friends, while parents are often more concerned about long‐term care issues:

The family might have a lot of care type issues and then the kid will be like, ‘Well I don't care about that, I want to access my computer, I want to be able to play with my friends’. And I think we need to be able to capture that, because the goals are different and that's fine. We might be able to achieve both the goals. (OT, interview)

Provided both the patient and family members are willing to negotiate a set of realistic goals, this is not considered problematic by the therapists. Indeed, in order to engage the patient as much as possible (as stipulated by the COPM) it is common for the therapists to negotiate a set of goals with a patient and then a separate set with the supporting family. In order to encourage patients to answer honestly and ensure they are not spoken for by their family, part of the goal‐setting session may be carried out with the patient alone. More often, though, this will simply involve instructing family members to wait their turn and not interrupt one another.

Importantly, while the COPM provides an overall script, it does not prescribe exactly how much of the work should be achieved. It therefore provides some flexibility, enabling the therapists to utilise their practice skills and experience‐informed dispositions. This flexibility constitutes a form of ‘power/discretion’: clinicians are accorded a space in the goal‐setting session within which they can decide which tools and techniques to assemble and deploy. Therapists may draw on their emotional work skills (Bolton 2001), for example, by managing their own emotions – and thus those of the patient and family members. The following example illustrates how PMDS therapists use humour to quell potentially disruptive tension. Toward the end of the session, Carl is visibly tired and his mother is beginning to answer on his behalf. In order to get Carl to provide an answer without being influenced by his mother, she received the following instruction:

OT Mum! Don't influence him! Hide your face and cover your ears, we need to hear from Carl!

The instruction was given at high volume but with obvious jest: the OT was smiling as she said it, and Carl along with the PT and psychologist responded with laughter. The instruction had the intended effect: Mum, also chuckling, turned her chair so that she was not facing Carl and covered her ears with her hands, enabling Carl to respond and to act as a spokesperson.

In addition to their professional and emotional resources, therapists may also choose to employ the use of communication aids to help patients act as spokespersons. The team members have devised a vision board that can be used to communicate with patients who are unable to communicate verbally or access an electronic aid. Depending on the age of the patient, the vision board will have a series of numbers, the words yes and no, happy and sad faces. As the therapy assistant explains:

We have a vision board where we put our numbers from one to ten for the importance of a certain goal … yes and no smiley face and sad face … Depends on how able they are … we have children who can easily move the upper limb and point. (Therapy assistant, interview)

These tools can be seen as what Latour refers to as intermediaries (Latour 2005: 39); entities that transport meaning or force from one agent (the patient) to another (the therapists), thus enabling the former to act (or influence) the latter. They constitute, then, the patient's constrained ‘power to’ engage in the session. In some cases, however, these tools may be insufficient. The severity of the child's affliction may prevent the use of such tools and their family members will speak on their behalf (although some attempt will be made to include the child):

If we're unable to have a whole sentence from the child or get an idea what they will want to achieve, then the parents set the goals and we have a yes/no conversation with the child. (Therapy assistant, interview)

Thus, during the goal‐setting session the COPM accords therapists with authority (‘power over’), and a degree of flexibility to draw on professional and emotional resources at hand, and to deploy additional communication aides (‘power/discretion’). These practices, along with the arranged, informal, unintimidating working space, help ensure that the patients and family members are able to act as spokespeople (constrained ‘power to’). In other words, they help ensure that the COPM script and its patient‐centred normative orientation are enacted in actual clinical practice, and they are all, therefore, implicated in the ethical work of negotiating expectations.

## Discussion: patient‐centredness, power and ethics

Patient‐centred medicine and patient empowerment have been heralded as a much‐needed rebalancing of the clinician–patient dynamic; as part of a movement away from the paternalism of the past towards a more egalitarian relationship. In the interest of anticipating the impact of the emerging patient‐centred and patient empowerment movement, and with the intention of encouraging deliberation on what impact the movement ought to have, we examined the role of patient‐centred tools in deploying and structuring power relations. Specifically we have used an ANT‐informed analytical perspective to illustrate how, in our PMDS case study, patient‐centred tools are implicated in establishing relations that constitute ‘power storage’, ‘power over’, ‘power to’ and ‘power/discretion’. We suggest that the COPM itself represents a form of ‘power storage’. The team's adoption of the tool into their routine clinical practice means that the power relations it prescribes are enacted again and again and thus become institutionally embedded. May *et al*. (2006) have referred to the institutionalisation of such patient‐centred tools as characteristic of an era of technogovernance. In this era, technological devices (such as decision‐making tools) are increasingly used as a means of governing the tensions in clinical settings; tensions that arise, for example, from the convergence of potentially conflicting ideas of evidence‐based medicine and patient‐centred medicine. Technogovernance, May *et al*. state, entails the prescribing of clinician practices so that patients are guided and their narratives are appropriately situated (May *et al*. 2006).

We have sought to explore precisely how such guiding and situating is actually achieved in an interaction. During the goal‐setting sessions patients and their families are encouraged to actively engage in the setting of goals for DBS and offer their perspectives and articulate those aspects of daily life that are most important to them. They are, in other words, encouraged to act as spokespersons (Latour 2005: 31). Yet to act as such requires a carefully configured space that contains the tools and resources through which the spokesperson can communicate in a meaningful way. In the PMDS goal‐setting session, this configuring of space is scripted by the COPM and it is directed by the therapist. The patients’ ‘power to’, therefore, is a consequence of the therapists’ degree of ‘power over’ them, as prescribed by the COPM. This of course aligns with Latour's argument that to be an agent is to be both affected by, and to affect, a wider assemblage of elements and agents (Latour, 2005). The striking difference between the positions accorded to the therapists and the patient by the COPM, however, is the degree to which the former possess ‘power/discretion’: the therapists have some flexibility to deploy various tools and professional capacities as they see fit, in order to configure the space that enables the latter to act as a spokesperson. Patients and supporting family members are, in comparison, provided very little ‘power/discretion’ except, perhaps, in choosing which aspects of daily life should be proffered as potential goals during the session. By using Law's (1991a) taxonomy of power as an analytical framework, we can see how patient‐centred interactions entail micro‐power dynamics that are asymmetrically distributed and considerably more complex than the more egalitarian models often mobilised by advocates of patient‐centredness.

We highlight this not to criticise the COPM tool nor to criticise the PMDS’ use of the tool. Such power relations may be ethically favourable in such a context, and indeed in most clinical contexts such power relations – particularly an asymmetrical distribution of ‘power/discretion’ – will be necessary for the completion of productive clinical work (such as a surgical procedure or a diagnosis). Indeed it can be argued that all important ethical work will be accomplished only by the sort of assembling and structuring of power relations we have discussed here, and that power and ethics should be more routinely analysed together rather than separately. More specifically, it is important to ensure that asymmetrical power dynamics in patient‐centred interactions are not elided by discourses that herald and overestimate the egalitarian aspects of patient‐centredness and patient empowerment.

Uncovering the way in which patient‐centred tools are implicated in structuring and managing power relations helps to open the movement up for more informed bioethical deliberation about what patient‐centredness and patient empowerment ought to look like in specific clinical contexts. There may be, for example, circumstances in which it is ethically favourable for patients and families to be accorded a much higher degree of ‘power/discretion’. In such a situation clinicians might adopt a position akin to what Latimer refers to (in her forthcoming book *Aging and Biopolitics at the Limits of Life*) as ‘careful science’, in which they adopt a sense of willingness to be affected by otherness, heterogeneity and uncertainty (Latimer 2014). We suggest that it is also particularly important to be attentive to circumstances that approximate something close to domination; undesirable circumstances that afford some actors considerable ‘power/discretion’ and ‘power over’ others. Latimer's account (2004) of how hospital consultants deploy various materials to assert their (medical) authority over other professionals in multidisciplinary settings is an example of this. There is therefore an important space for bioethicists and other interested parties to develop arguments about, and to debate, the patient‐centred values and principles that ought to be enacted in various biomedical contexts and practices. However, if such arguments are to have any applied relevance, they will need to be informed by a grounded consideration of the process through which such values and principles are achieved or undermined, including how they may be promoted or constrained by non‐human elements.
